# TRENDS AND CHARACTERISTICS OF WORKING CAREGIVERS USING PAID FAMILY LEAVE PROGRAMS IN RHODE ISLAND AND WASHINGTON

**DOI:** 10.1093/geroni/igae098.3912

**Published:** 2024-12-31

**Authors:** Jennifer Im, Edwin Wong, Paul Fishman, Heather Hill, Bryan Weiner

**Affiliations:** University of Washington, Seattle, Washington, United States; University of Washington, University of Washington, Washington, United States; University of Washington, University of Washington, Washington, United States; University of Washington, Seattle, Washington, United States; University of Washington, Seattle, Washington, United States

## Abstract

Many family caregivers are part of the workforce and integral to older adults’ care. State Paid Family Leave (PFL) programs offer partial wage replacement to working caregivers. However, data on caregiving leave use is limited. This study examines trends and characteristics of workers who sought caregiving leave in Rhode Island (RI) and Washington (WA) using PFL program administrative data. Outcomes were application and approval for caregiving leave. Covariates included age, sex, ethnicity (WA only), income, primary language, and industry. Logit models with average marginal effects were used. In RI, 14,323 claims were recorded between 2015-22; 82.3% were approved. In WA, 75,739 claims were recorded between 2020-23; 76.7% were approved. Overall, caregiving applications increased annually. Applicants’ mean ages were mid-to-late 40s (RI=48.3; WA=46.9). Most applicants were female (RI=68.3%; WA=61.5%) and English-speaking (RI=95.1%; WA=89.2%). WA applicants were predominantly White (57.5%), Hispanic (13.8%), and mixed race (8.3%). Applications for spousal caregiving was most common in RI (36.3%); caregiving for children was most common in WA (39.1%). The lowest-earning workers had the highest percentage of denied claims (RI=35.5%; WA=29.8%). In both states, older age and higher income were significantly associated with claim approval. In RI, worker caring for spouse, parent, or grandparent had significantly lower probabilities of claim approval, compared to children; In WA, the opposite was observed. The results suggest caregiving PFL is rising among workers, with claim approval more likely for older workers. Higher income was associated with claim approval suggesting programs may not benefit workers that need it the most.

